# Carriage and Acquisition of Extended-spectrum β-Lactamase–producing Enterobacterales Among Neonates Admitted to Hospital in Kilifi, Kenya

**DOI:** 10.1093/cid/ciy976

**Published:** 2019-03-04

**Authors:** Ngure Kagia, Patrick Kosgei, Michael Ooko, Leonard Wafula, Neema Mturi, Kirimi Anampiu, Salim Mwarumba, Patricia Njuguna, Anna C Seale, James A Berkley, Christian Bottomley, J Anthony G Scott, Susan C Morpeth

**Affiliations:** 1 Kenya Medical Research Institute–Wellcome Trust Research Programme, Centre for Geographic Medicine Research- Coast, Kilifi; 2 Nuffield Department of Clinical Medicine, University of Oxford, United Kingdom; 3 Department of Infectious Disease Epidemiology, London School of Hygiene and Tropical Medicine, United Kingdom; 4 Counties Manukau District Health Board, Auckland, New Zealand

**Keywords:** neonates, extended-spectrum β-lactamase, carriage, acquisition, risk factors

## Abstract

**Background:**

Infections caused by extended-spectrum β-lactamase–producing Enterobacterales (ESBL-E) among hospitalized neonates in sub-Saharan Africa pose significant clinical challenges. Data on prevalence and acquisition of ESBL-E carriage among hospitalized neonates in the region are few, and risk factors for transmission are not clearly defined.

**Methods:**

In a cohort study of consecutive neonatal admissions to Kilifi County Hospital from July 2013 through August 2014, we estimated ESBL-E carriage prevalence on admission using rectal swab cultures and identified risk factors using logistic regression. Using twice-weekly follow-up swabs, we estimated the incidence and identified risk factors for ESBL-E acquisition in hospital using Poisson regression.

**Results:**

The prevalence of ESBL-E carriage at admission was 10% (59/569). Cesarean delivery, older neonatal age, and smaller household size were significant risk factors. Of the 510 infants admitted without ESBL-E carriage, 238 (55%) acquired carriage during their hospital stay. The incidence of acquisition was 21.4% (95% confidence interval, 19.0%–24.0%) per day. The rate was positively associated with the number of known neonatal ESBL-E carriers and with the total number of neonates on the same ward.

**Conclusions:**

Carriage of ESBL-E was common among neonates on admission, and in-hospital acquisition was rapid. The dissemination and selection of ESBL-E appears to be driven by hospital exposures, operative delivery, and neonatal ward patient density. Further attention to infection control, patient crowding, and carriage surveillance is warranted.

## Teaser

We report a prospective hospital-based longitudinal study that estimates the extended-spectrum β-lactamase–producing Enterobacterales (ESBL-E) carriage prevalence among neonates on admission, the incidence of acquisition of ESBL-E carriage in hospital, and the risk factors for neonatal prevalent and incident ESBL-E carriage.

Infection and carriage rates of extended-spectrum β-lactamase–producing Enterobacterale*s* (ESBL-E) are on the rise globally and pose a particular threat to neonates [1–3]. Outbreaks of multidrug-resistant infections due to ESBL-E in hospitals are common [4–7] and are a growing burden, especially among neonates [3].

It is known that neonatal ESBL-E carriage can be a precursor to invasive infections [7, 8], but the epidemiology of transmission in sub-Saharan Africa is poorly characterized. In sub-Saharan Africa, data on neonatal ESBL-E infection and carriage are scarce [2, 3], but there is some evidence of hospital-acquired carriage in older children. In a general pediatric ward in Madagascar, prevalence of carriage of ESBL-E in stool was found to be 21% on admission and 57% on discharge among patients discharged ≥48 hours after admission [9]. In the community, among children and adults in Madagascar [10], prevalence of ESBL carriage was 10%.

At Kilifi County Hospital (KCH), we have observed sporadic outbreaks of ESBL-E bacteremia among neonatal admissions over several years. These infections often have a poor outcome (in KCH, the case-fatality risk for hospital-acquired pediatric bloodstream infections is 54% [11]). We have also observed an increase in the proportion of ESBL-producing invasive *Klebsiella pneumoniae* over a decade at KCH [12].

Here we report a prospective, hospital-based, longitudinal study at KCH to estimate ESBL-E carriage prevalence among neonates on admission, the incidence of acquisition of ESBL-E carriage in hospital, and the risk factors for neonatal prevalent and incident ESBL-E carriage.

## MATERIALS AND METHODS

### Study Design and Sampling Procedure

Neonatal admissions were eligible for recruitment into the study if they were admitted to the high-dependency unit between 1 July 2013 and 29 August 2014 or to the neonatal rooms in the general pediatric ward between 16 August 2013 and 29 August 2014. The high-dependency unit consists of an open ward with 6 beds for older children and 2 small rooms for neonatal admissions. The neonatal rooms are of equal size and have a combined bed capacity of 8. In the general pediatric ward, there are 2 neonatal rooms with a combined bed capacity of 24, including 5 incubators, 4 small beds, and 15 cots. KCH practices comprehensive obstetric care, as defined by the World Health Organization, with cesarean delivery services available.

### Data and Clinical Sample Collection

Epidemiological and clinical data were collected on admission and entered in real time into an electronic medical record system. Rectal swabs were collected on admission (day 0), at days 2, 4, and 6, and twice weekly thereafter until an ESBL-E was isolated or until hospital discharge or death, whichever came first. Rectal swabs were collected using premoistened viscose-tipped swabs and placed in Amies transport media (Deltalab, Barcelona, Spain). The number of neonates in each room, bed location, and antimicrobial use of all participants was recorded daily. Blood culture is performed routinely at admission on all children hospitalized at KCH [13]. Clinical samples are collected at the discretion of the attending clinician.

### Laboratory Procedure

Rectal swabs were inoculated onto 5% horse blood agar and MacConkey agar supplemented with 8% gentamicin. Cefotaxime (30 μg) and ceftazidime (30 μg) antibiotic disks (Oxoid, United Kingdom) were added on the second and fourth streaking zones on the blood agar plate to detect bacteria resistant to third-generation cephalosporins. Blood agar plates were incubated in a carbon dioxide incubator while MacConkey agar plates were incubated in an aerobic incubator for 24 hours at 35°C ± 2°C. Oxidase-negative, gram-negative rods were subcultured and identified using standard techniques (API 20E, bioMérieux, France). Antimicrobial susceptibility testing was performed using the disk diffusion method according to the Clinical and Laboratory Standards Institute 2014 guidelines [14]. ESBL testing was performed for isolates that were nonsusceptible to third-generation cephalosporins using the double disk method [14]. External quality assurance was provided for by the UK National External Quality Assessment service.

### Analysis

The binomial confidence interval (CI) around a prevalence estimate is widest (for a fixed sample size) when the estimate is 50%. We calculated that a sample size of 555 neonates would be required to estimate a 50% prevalence of ESBL-E carriage with a precision of ±5%.

**Figure 1.**
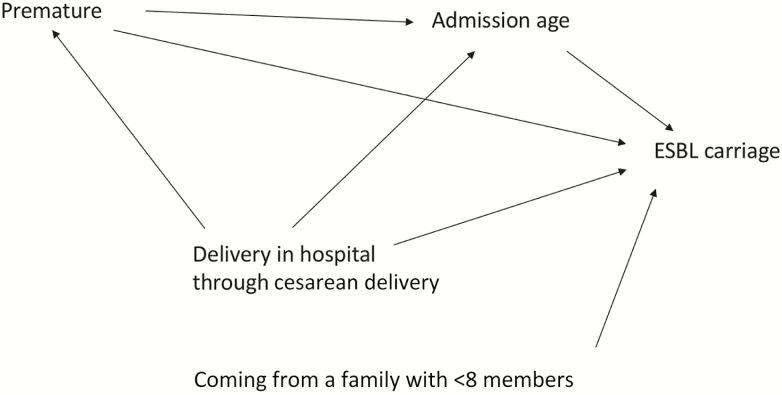
Causal diagram for determinants of extended-spectrum β-lactamase–producing Enterobacterales carriage at admission. Only variables that had plausible interactions as shown in the causal diagram were included in the multivariable model. Abbreviation: ESBL, extended-spectrum β-lactamase.

Carriage on admission (prevalent carriage) was defined as a positive culture on the first rectal swab, provided it was obtained within 48 hours of admission. Logistic regression was used to determine risk factors for carriage at admission. The factors considered were infant age, sex, maternal age, infant weight at admission, current method of feeding, place and mode of delivery, prematurity (≤37 weeks’ gestation, estimated by the admitting clinician), number living in the same household, type of toilet, and main source of water. Multivariable logistic regression models were fitted after confounders had been identified (Figure 1). Infant age at admission was adjusted for prematurity and place/mode of delivery, and prematurity was adjusted for age on admission.

Kaplan-Meier curves were used to describe the time to acquisition of carriage in hospital among neonates who did not have ESBL-E carriage at admission. In this analysis, follow-up time, which was measured in days after admission, was censored at the earliest of (1) time of the first ESBL-E culture-positive swab, or (2) time of last swab collection if the neonate died or was discharged and had remained negative throughout the course of admission. For neonates who acquired ESBL-E, the date of acquisition was assumed to be the midpoint between the date of the last negative swab and the date of the first positive swab.

We calculated the rate of ESBL-E acquisition per 100 days at risk. Poisson regression was used to identify predictors of the acquisition rate and to test for interactions. The potential predictors were both time-invariant (eg, weight at admission, place/mode of delivery, mother’s age, and age at admission) and time-varying (eg, number of known ESBL carriers on the ward). A multivariable Poisson regression model was fitted to investigate the effect of crowding on ESBL-E acquisition. The model included as covariates the ward, the number of neonates on the ward, and the number of known ESBL-E carriers on the ward.

We defined multidrug resistance as resistance to at least 1 agent in 3 or more antimicrobial categories [15]. Statistical analyses were done with Stata version 12.0 software (StataCorp, College Station, Texas).

### Ethical Considerations

The Kenya Medical Research Institute National Ethical Review Committee approved the study (SSC 2301). Informed consent was obtained from all parents/guardians before enrollment.

## RESULTS

**Figure 2.**
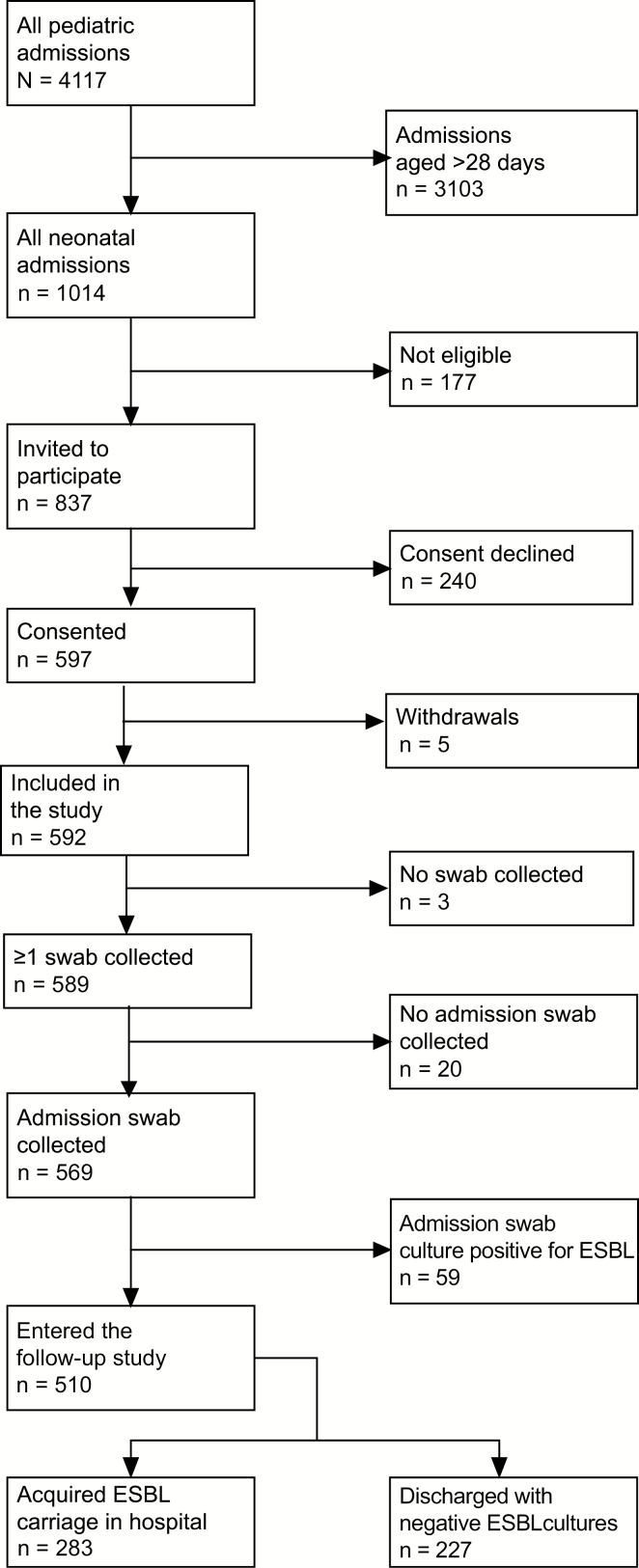
Flow of subjects recruited in the study. Among the 177 ineligible neonatal admissions, 57 were admitted to the general pediatric ward before the study rolled out in that ward; 42 died before consenting; 30 were not consented because there was no competent adult available to provide consent on behalf of the child within the first 48 hours of admission; 21 were discharged on admission; 5 were cases of readmission and their extended-spectrum β-lactamase Enterobacterales carriage status was known; 5 were not followed up after admission; 4 absconded and could not be traced after admission; 4 were considered too tiny for a sample to be collected; 3 were admitted for elective surgery and consenting was not done; 2 had congenital abnormalities and were not approached to participate; 2 could not be enrolled because the parent was a minor; 1 was not consented because the mother could not be approached for consent; and 1 was enrolled after recruitment had stopped. Abbreviation: ESBL, extended-spectrum β-lactamase.

During the study period, 1014 neonates were admitted to KCH, and the parents/guardians of 597 neonates gave consent for them to participate in the study (Figure 2). The median age of the participants was 1 day (interquartile range [IQR], 0–3 days) and the median duration of hospital stay was 5 days (IQR, 3–9 days).

Of 597 who gave consent to participate, 5 parents/guardians withdrew consent and 23 neonates had no swab collected. The prevalence of ESBL carriage at admission was 10% (59/569). From the 59 neonates with ESBL-E carriage at admission, there were 65 isolates consisting of 31 *Klebsiella pneumoniae*, 25 *Escherichia coli*, 8 *Enterobacter cloacae*, and 1 *Klebsiella oxytoca*. Multiple colonization, that is, colonization with ≥2 ESBL-E isolates from 1 participant, was found in 6 of 59 neonates (10%).

**Figure 3.**
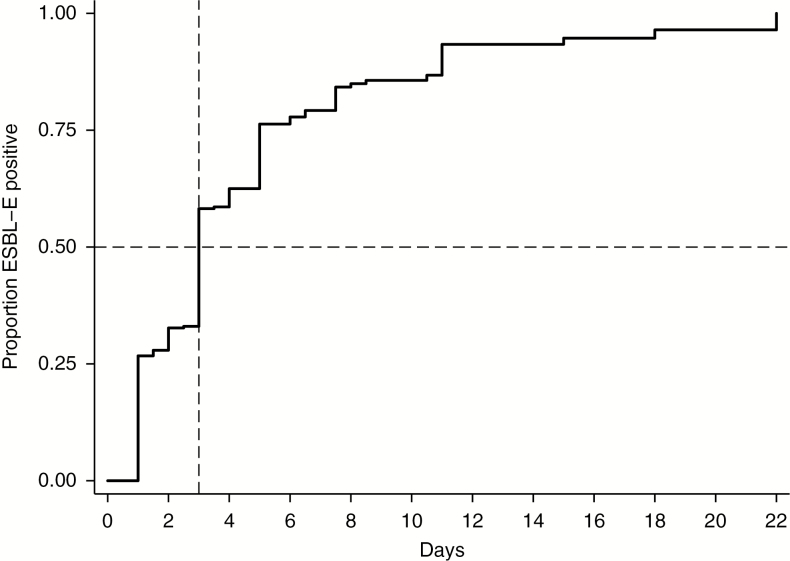
Kaplan-Meier estimate of extended-spectrum β-lactamase–producing Enterobacterales (ESBL-E) carriage acquisition as a function of days after admission among 510 neonates who were ESBL-E carriage negative at admission. Abbreviation: ESBL-E, extended-spectrum β-lactamase Enterobacterales.

Among the 510 noncarriers on admission, 55% (283/510) acquired ESBL-E during their hospital stay. The incidence of ESBL-E acquisition was 21.4 (95% CI, 19.0–24.0) per 100 child-days of observation, and the median time to acquisition among these patients was 3 (IQR, 1–5) inpatient days (Figure 3). Nine neonates were diagnosed with ESBL-E bacteremia during this study, all of whom had ESBL-E isolated from fecal carriage prior to or on the same day as blood was collected.

Most ESBL-E isolates were multidrug resistant, with resistance to chloramphenicol, trimethoprim-sulfamethoxazole, quinolones, and gentamicin being common; none were resistant to imipenem and only 5% were resistant to amikacin (Table 1).

**Table 1.** Nonsusceptibility Profile for *Klebsiella pneumoniae, Escherichia coli*, and *Enterobacter cloacae* Carriage Isolates

Antibiotic Tested	Timing of Admission	*Klebsiella pneumoniae*		*Escherichia coli*		*Enterobacter cloacae*	
	On Admission^a^	n = 31		n = 25		n = 8	
	After Admission^b^	n = 196		n = 73		n = 42	
Chloramphenicol	On admission	11	(35.5)	3	(12.0)	7	(87.5)
	After admission	66	(33.7)	17	(23.3)	33	(78.6)
Ciprofloxacin	On admission	13	(41.9)	16	(64.0)	5	(62.5)
	After admission	99	(50.5)	64	(87.7)	25	(59.5)
Cotrimoxazole	On admission	31	(100)	22	(88.0)	7	(87.5)
	After admission	193	(98.5)	70	(95.9)	38	(90.5)
Gentamicin	On admission	30	(96.8)	11	(44.0)	7	(87.5)
	After admission	192	(98.0)	58	(79.5)	38	(90.5)
Amikacin	On admission	2	(6.5)	1	(4.0)	0	(0)
	After admission	19	(9.7)	3	(4.1)	2	(4.8)
Imipenem	On admission	0	(0)	0	(0)	0	(0)
	After admission	0	(0)	0	(0)	0	(0)

Data are presented as No. (%). All isolates were extended-spectrum β-lactamase–producing Enterobacterales.

^a^On admission: ≤48 hours after admission.

^b^After admission: >48 hours after admission.

### Risk Factors for ESBL-E Carriage at Admission

In the univariable analysis, variables associated with prevalent ESBL-E carriage on admission were being born at term, older infant age at admission, having <8 people living in the same house, and hospital delivery, particularly by cesarean delivery (Table 2 and Supplementary Table 1). Babies born prematurely were more commonly admitted directly to the neonatal ward (107/157 [68%]) than babies born at full term (114/410 [28%]; *P* < .001). After adjusting for prematurity and place/mode of delivery, increasing infant age was positively associated (*P* < .001) with ESBL-E carriage at admission, with odds ratios (ORs) of 1.72 (95% CI, .69–4.27) and 3.88 (95% CI, 1.47–10.21) among neonates aged 1–2 days and 3–28 days, respectively, relative to the odds of ESBL carriage among neonates admitted on the day of birth. Being born at term was not associated with ESBL-E carriage after adjusting for the effect mediated through age on admission. We did not estimate an adjusted OR for place/mode of delivery and number of people in the same household, as the associations were not confounded (Figure 1).

**Table 2.** Univariable Analysis of Risk Factors for Extended-spectrum β-Lactamase–producing Enterobacterales Colonization at Admission

Variable	no./No.	(%)	OR	(95% CI)	*P* Value
Prematurity					.017
No	50/410	(12.2)	1	…	
Yes	9/157	(5.7)	0.44	(.21–.91)	
Weight at admission					.083
<2.5 kg	20/250	(8.0)	1	…	
≥2.5 kg	39/313	(12.5)	1.64	(.93–2.89)	
Sex					.327
Male	31/333	(9.3)	1	…	
Female	28/236	(11.9)	1.31	(.76–2.25)	
Age at admission, d					<.001
0	8/221	(3.6)	1	…	
1–2	16/177	(9.0)	2.65	(1.11–6.33)	
3–28	35/171	(20.5)	6.85	(3.09–15.21)	
Place/mode of delivery					.002
Community	6/116	(5.2)	1	…	
Hospital non-CS	32/339	(9.4)	1.91	(.78–4.69)	
Hospital CS	21/105	(20.0)	4.58	(1.77–11.86)	
Mother’s age, y					.780
<18	6/54	(11.1)	1	…	
18–35	47/455	(10.3)	0.92	(.37–2.27)	
>35	4/53	(7.6)	0.65	(.17–2.46)	
Main water source					.153
Tap in the compound	27/179	(15.1)	1	…	
Tap in community	24/245	(9.8)	0.61	(.34–1.10)	
Borehole in community	6/54	(11.1)	0.70	(.27–1.81)	
Natural source	1/33	(3.0)	0.18	(.02–1.34)	
Water vendor	1/18	(5.6)	0.33	(.04–2.59)	
Current feeding method					.251
Breastfeeding	39/315	(12.4)	1	…	
No breastfeeding	17/187	(9.1)	0.71	(.39–1.29)	
Type of toilet					.169
Toilet in house	16/122	(13.1)	1	…	
Toilet shared in compound/ community	37/333	(11.1)	0.83	(.44–1.55)	
None	6/101	(5.9)	0.42	(.16–1.11)	
No. of people living in the same house					.012
1–4	29/227	(12.8)	1	…	
5–7	22/167	(13.2)	1.04	(.57–1.88)	
8–40	8/162	(4.9)	0.35	(.16–.80)	

“At admission” indicates that swabs were collected within 48 hours after admission. Missing data: prematurity (n = 2), weight at admission (n = 6), place/mode of delivery (n = 9), mother’s age (n = 7), main water source (n = 40), current feed (n = 67), type of toilet (n = 13), number of people living in the same house (n = 13).

Abbreviations: CI, confidence interval; CS, cesarean delivery; OR, odds ratio.

### Risk Factors Associated With Acquisition of ESBL-E During Hospitalization

In the univariable analysis, hospital ward on admission, the number of neonates present in the hospital, the number of other neonates admitted in the same ward, and the number of known ESBL-E carriers were associated with incident acquisition of carriage (Table 3). Both current number of known neonatal ESBL-E carriers and number of other neonates in the same ward were positively associated with carriage acquisition when simultaneously included in the multivariable model (Table 4), and there was no interaction between these risk factors. In both the univariable and multivariable analyses, the number of neonates in the ward exhibited a threshold effect whereby there was a plateau effect in carriage acquisition beyond 10 patients per ward. Recorded antibiotic prescription, specifically use of third-generation cephalosporins during the inpatient stay, was not shown to be associated with ESBL-E acquisition.

**Table 3.** Univariable Analysis of Risk Factors for Acquisition of Extended-spectrum β-Lactamase–producing Enterobacterales Colonization in Hospital

Variable	Events	Person-Days	Rate^a^	Rate Ratio	(95% CI)	*P* Value
Prematurity						.948
No	171	802.5	21.31	1	…	
Yes	112	521.5	21.48	1.01	(.79–1.28)	
Weight at admission						.979
<2.5 kg	153	709	21.58	1	…	
≥2.5 kg	126	582	21.65	1.00	(.79–1.27)	
Sex						.155
Male	168	731	22.98	1	…	
Female	115	594	19.36	0.84	(.66–1.07)	
Age at admission, d						.769
0	127	622	20.42	1	…	
1–2	82	364	22.53	1.10	(.84–1.46)	
3–28	74	339	21.83	1.07	(.80–1.42)	
Place/mode of delivery						.185
Community	74	354	20.90	1	…	
Hospital, non-CS	160	769	20.81	1.00	(.76–1.31)	
Hospital, CS	47	166.5	28.23	1.35	(.94–1.95)	
Mother’s age, y						.583
<18	27	146.5	18.43	1	…	
18–35	228	1060	21.51	1.17	(.78–1.74)	
>35	25	102	24.51	1.33	(.77–2.29)	
Treated with antibiotics						.566
No	20	82.5	24.24	1	…	
Yes	263	1242.5	21.17	0.87	(.55–1.38)	
Treated with ampicillin and gentamicin						.403
No	35	143	24.48	1	…	
Yes	248	1182	20.98	0.86	(.60–1.22)	
Treated with third-generation cephalosporins						.44
No	206	937	21.99	1	…	
Yes	77	388	19.85	0.90	(.69–1.17)	
Duration of antibiotic use, d						.627
1–3	24	133.5	17.98	1	…	
4–7	135	624	21.63	1.20	(.78–1.86)	
>7	104	468	22.22	1.24	(.79–1.93)	
No. of neonates present in the hospital per day						.001
1–19	71	465.5	15.25	1	…	
20–32	163	662	24.62	1.61	(1.22–2.13)	
33–45	49	197.5	24.81	1.63	(1.13–2.34)	
No. of known ESBL-E carriers per day						<.001
0–4	22	260.5	8.45	1	…	
5–9	88	477	18.45	2.18	(1.37–3.49)	
10–14	122	407	29.98	3.55	(2.25–5.59)	
15–21	51	180.5	28.25	3.35	(2.03–5.52)	
No. of neonates on the same ward						<.001
0–4	14	146	9.59	1	…	
5–9	49	357	13.73	1.43	(.79–2.59)	
10–14	70	282.5	24.78	2.58	(1.46–4.59)	
15–20	81	290	27.93	2.91	(1.65–5.14)	
21–29	69	249.5	27.66	2.88	(1.62–5.12)	
Ward at admission						.003
HDU	45	302	14.90	1	…	
General pediatric	236	1004	23.51	1.58	(1.15–2.17)	
Current feeding method						.599
Breastfeeding	140	661.5	21.16	1	…	
No breastfeeding	111	490.5	22.63	1.07	(.83–1.37)	
Type of toilet						.943
Toilet in house	57	248	22.98	1	…	
Toilet shared with community	162	733	22.10	0.96	(.71–1.30)	
None	59	255.5	23.09	1.00	(.70–1.45)	
Main water source						.327
Tap in compound	87	328.5	26.48	1	…	
Tap in community	124	554	22.38	0.85	(.64–1.11)	
Borehole in community	26	141	18.44	0.70	(.45–1.08)	
Natural source	19	84.5	22.49	0.85	(.52–1.39)	
Water vendor	7	47	14.89	0.56	(.26–1.21)	
No. of people living in the same house						.227
1–4	112	440	25.45	1	…	
5–7	85	425.5	19.98	0.78	(.59–1.04)	
8–40	81	371	21.83	0.86	(.64–1.14)	

Abbreviations: CI, confidence interval; CS, cesarean delivery; ESBL-E, extended-spectrum β-lactamase Enterobacterales; HDU, high-dependency unit.

^a^Rate per 100 person-days.

**Table 4.** Multivariable Analysis of Risk Factors for Acquisition of Extended-spectrum β-Lactamase–producing Enterobacterales Colonization in Hospital

Risk Factor	Rate Ratio	(95% CI)	*P* Value
No. of known ESBL-E carriers^a^			
0–4	1		<.001
5–9	1.77	(1.09–2.88)	
10–14	2.80	(1.70–4.59)	
15–21	2.57	(1.48–4.48)	
No. of neonates on the same ward^b^			.025
0–4	1		
5–9	1.35	(.74–2.46)	
10–14	2.13	(1.16–3.90)	
15–20	1.90	(1.04–3.48)	
21–29	1.76	(.93–3.31)	

Abbreviations: CI, confidence interval; ESBL-E, extended-spectrum β-lactamase Enterobacterales.

^a^Rate ratio adjusted for number of known ESBL-E carriers and ward at admission.

^b^Rate ratio adjusted for number of neonates on the same ward and ward at admission.

## DISCUSSION

Our study reveals that among neonates admitted to a rural Kenyan hospital, 10% were already carriers of ESBL-E. Among those neonates who were not carriers at admission, 21.4% acquired ESBL-E carriage each day of admission; thus, more than half of the neonates were colonized with ESBL within the first 3 inpatient days.

For babies coming in to hospital, the main risk factors for existing rectal carriage with ESBL-E were cesarean delivery in hospital and older infant age at admission. For those admitted without carriage of ESBL-E, the principal risk factors for acquisition in hospital were the number of other neonates in the ward and the number with ESBL-E carriage.

Delivery through cesarean delivery has been reported to be a significant risk factor for prolonged fecal colonization with ESBL-producing *K. pneumoniae* [16] and also a determinant of intestinal microflora early in life [17, 18]. Mothers undergoing cesarean delivery are treated with antibiotics for surgical prophylaxis, sometimes extended to treatment of wound infections [19], which may select for antibiotic-resistant enteric bacteria. In Cambodia, young hospital-born infants were found to be at a greater risk of early colonization by third-generation cephalosporin-resistant gram-negative rods compared to infants born at home, a health center, or other locations and subsequently admitted to hospital [20]. In Madagascar, Herindrainy et al reported that low birth weight, cesarean delivery, and use of antibiotics by mothers at delivery were independently associated with neonatal acquisition of ESBL-E during the first month of life [21].

The finding that babies coming from a large family of >8 household members were less likely to carry ESBL-E at admission was surprising. We speculate that these neonates may have a more diverse gut microbiome, which could be protective against acquisition of ESBL-E carriage. Increased neonatal age at hospital admission was associated with a greater likelihood of ESBL-E carriage, as expected, since older neonates have had more time to acquire carriage.

Overall, we isolated ESBL-E from 10% of swabs within 48 hours of admission. In a cross-sectional ESBL-E carriage study done in a Tanzanian hospital, the overall neonatal prevalence of ESBL-E carriage was 25.4% [22]. Our findings suggest that some acquisition occurs before neonates come into the pediatric wards; we can speculate that this does not only come from their mothers but also from the procedures and settings of childbirth, particularly cesarean delivery. We did not collect data on ESBL-E carriage in mothers or maternal antibiotic use.

Among neonates admitted without carriage, 55% acquired ESBL-E during hospitalization. An ESBL-E carriage study in a tertiary hospital in Rwanda among inpatients of all ages reported that 55% of participants acquired ESBL-E carriage during hospitalization [23]. A study in Madagascar reported that 48% of pediatric noncarriers at admission acquired ESBL-E during hospitalization.

Our findings from the longitudinal study suggest that the greatest risk factors for ESBL-E acquisition in hospital were having increased numbers of existing ESBL-E carriers among the neonatal patients and a greater number of neonates admitted to the ward. We assume that having increased numbers of ESBL-E carriers increases the opportunity for transmission. This finding corresponds with a prospective cohort study done in the general intensive care unit of a hospital in Greece; colonization pressure contributed significantly to acquisition of carriage of carbapenemase-producing *Klebsiella pneumoniae* in hospital [24]. Intuitively, hospital crowding is expected to be associated with higher rates of ESBL-E transmission and our results support this. However, our findings suggest a threshold effect where risk plateaued after admitting >10–14 neonates in a ward, suggesting that transmission effects associated with crowding are complex. Restricting the number of neonatal admissions to the hospital is impractical, but this does justify allocating increased space to neonatal admissions. Fixed low healthcare staff numbers relative to numbers of patients, the cultural practice of mothers caring for each other’s babies on the ward, physical proximity of adjacent neonates, and shared hygiene facilities may all contribute to acquisition of ESBL-E carriage by neonates in hospital. As well as direct transmission between babies on the ward, nosocomial carriage acquisition directly from the hospital environment is also possible. A study in Cambodia of transmission of third-generation cephalosporin-resistant *K. pneumoniae* isolates in a newly opened neonatal unit found that most clusters were likely to have been due to patient sources while 2 of 9 clusters could have been due to either an environmental or a patient source [25].

During the study period, 9 neonates were diagnosed with ESBL-E bacteremia. Nosocomial spread of ESBL-E carriage may result in outbreaks of ESBL-E bacteremia in the hospital; such outbreaks have occurred in KCH in recent years [12], signifying the importance of awareness of ESBL-E carriage. There is potential for surveillance to help inform hospital infection control and to assist in averting such outbreaks. At KCH, screening for carriage of ESBL-E among neonates is not routinely done, hand-washing facilities frequently lack water supply, and there are no fully dedicated infection control staff.

Antibiotic use has been shown to affect the composition of gut microbiota and is associated with ESBL-E carriage and acquisition [9, 16, 17, 24, 26]. Antimicrobial stewardship services are used as part of hospital infection control services to reduce ESBL carriage in well-resourced hospitals. We were unable to detect antibiotic use as a risk factor for ESBL-E acquisition in our study. We suspect that this is mainly attributed to the fact that 93% of our participants were given antibiotics during their hospital stay and we were therefore underpowered to observe any differences (Supplementary Table 2).

We did not collect data from babies after they were discharged from the hospital, but patients discharged with ESBL-E carriage have been shown to spread these ESBL-E within family units and close contacts [16, 27]. In a prospective cohort study of infants and their families in Norway, the median carriage duration among infants discharged with carriage of ESBL-producing *K. pneumoniae* after a hospital outbreak was 12.5 months [16]. If carriage of ESBL-E persists and intrahousehold transmission occurs, discharged patients may act as reservoirs of ESBL-E in the community.

Being a hospital-based study, focusing on sick newborns, our estimates of ESBL-E prevalence at admission cannot be generalized to community prevalence. It is theoretically possible that low-level ESBL-E carriage was more prevalent at admission than we were able to determine—below the detection rate by culture methods, but then amplified by selection pressure from the use of antibiotics in hospital until detectable. We also were only able to recruit 68% of eligible neonates, limiting the generalizability of our findings (Supplementary Table 1). Of note, significantly more parents/guardians of older neonates and of neonates born in hospital by cesarean delivery declined to participate in the study, suggesting that our estimate of prevalence of ESBL-E carriage at admission is likely to be an underestimate. Our prevalence and incidence estimates may also be underestimates as stool culture may be more sensitive than rectal swab culture; a single sample is less sensitive than multiple samples for culture; and some *Enterobacter* species, which are known to produce *AmpC* β-lactamases, may have tested false-negative for ESBL by the phenotypic method used. We did not find any carbapenem-resistant Enterobacterales (CRE) in this study, but it is known that such isolates are present in Kenya [28, 29]. Use of central quality-assured microbiology laboratories in surveillance for ESBL-E carriage could therefore be expected to have the added benefit of an early warning system for the introduction of CRE carriage.

In conclusion, our findings reveal a high incidence of ESBL-E colonization, which is endemic in this setting, among hospitalized neonates. Further work to investigate the association between ESBL-E acquisition and both cesarean delivery and crowding, perhaps including restrictions on room capacity and more deliberate cohorting of older neonates and those born in hospital through cesarean delivery, is needed. Given the link between ESBL-E carriage and outbreaks of potentially fatal ESBL-E infection, our data emphasize the importance of routine surveillance and hospital infection control.

## Supplementary Data

Supplementary materials are available at *Clinical Infectious Diseases* online. Consisting of data provided by the authors to benefit the reader, the posted materials are not copyedited and are the sole responsibility of the authors, so questions or comments should be addressed to the corresponding author.

ciy976_suppl_Supplementary_Table_S1

## Notes

***Acknowledgments.*** This study is published with the permission of the director of the Kenya Medical Research Institute, Nairobi. The authors thank Irene Mutuku and John Ojal for their help in setting up the study, all of the participants, and the Kilifi County Hospital management, clinical team, and laboratory staff for their involvement in this work.

***Financial support.*** This work was supported by the Medical Research Council/Department for International Development/Wellcome Trust–Joint Global Health Trials scheme (grant number MR/M007367/1 to J. A. B.) and the Wellcome Trust (grant numbers 098532 to J. A. G. S. and 093804 and 205184 to A. C. S.).

***Potential conflicts of interest.*** S. C. M. has received grants paid to her institution from the Bill & Melinda Gates Foundation and the Wellcome Trust. All other authors report no potential conflicts. All authors have submitted the ICMJE Form for Disclosure of Potential Conflicts of Interest. Conflicts that the editors consider relevant to the content of the manuscript have been disclosed.
