# Supplementary material for: Carriage and Acquisition of Extended-spectrum β-Lactamase–producing Enterobacterales Among Neonates Admitted to Hospital in Kilifi, Kenya
Source: Clin Infect Dis. 2019 Mar 4;69(5):751–9. doi: 10.1093/cid/ciy976 (PMC6695508; doi:10.1093/cid/ciy976)
Supplement: ciy976_suppl_Supplementary_Table_S1 [file ciy976_suppl_supplementary_table_s1.docx]

**Supplementary Material**

**Carriage and acquisition of Extended Spectrum Beta-Lactamase producing *Enterobacteriaceae* among neonates admitted to hospital in Kilifi, Kenya**

Ngure Kagia^1*^, Patrick Kosgei^1^, Michael Ooko^1^, Leonard Wafula^1^, Neema Mturi^1^, Kirimi Anampiu^1^, Salim Mwarumba^1^, Patricia Njuguna^1^, Anna C. Seale^1, 2, 3^, James A. Berkley^1, 2^, Christian Bottomley^3^, J. Anthony G. Scott^1, 3^, Susan C. Morpeth^1, 3, 4^

Affiliations

1. KEMRI- Wellcome Trust Research Programme, CGMR-Coast
2. Nuffield Department of Clinical Medicine, University of Oxford, Oxford, UK
3. London School of Hygiene and Tropical Medicine, London, UK
4. Counties Manukau District Health Board, Auckland, New Zealand

**Table S1. Characteristics of study neonates and those who were eligible but not included, either because they were not swabbed, or because they withdrew prior to the start of the study, or because their parents did not provide consent.**

| **Characteristic** | **Included in study**  **(N=569)** | | **Excluded from study (N=268)** | | **P - value** |
| --- | --- | --- | --- | --- | --- |
|  | **n** | **%** | **n** | **%** |  |
| **Sex** |  |  |  |  | 0.695 |
| Male | 333 | 58.5 | 153 | 57.1 |  |
| Females | 236 | 41.5 | 115 | 42.9 |  |
| **Age at admission** |  |  |  |  | 0.454 |
| 0 days | 221 | 38.8 | 93 | 34.7 |  |
| 1-2 days | 177 | 31.1 | 93 | 34.7 |  |
| 3-28days | 171 | 30.1 | 82 | 30.6 |  |
| **Weight at admission** |  |  |  |  | **0.026** |
| <2.5kgs | 250 | 44.4 | 96 | 36.2 |  |
| ≥2.5kgs | 313 | 55.6 | 169 | 63.8 |  |
| **Prematurity** |  |  |  |  | **0.004** |
| Yes | 157 | 27.7 | 49 | 18.5 |  |
| No | 410 | 72.3 | 216 | 81.5 |  |
| **Place/mode of delivery** |  |  |  |  | **0.005** |
| Community | 116 | 20.7 | 30 | 11.4 |  |
| Hospital, non-CS | 339 | 60.5 | 175 | 66.5 |  |
| Hospital, CS | 105 | 18.8 | 58 | 22.1 |  |
| **Mother’s age** |  |  |  |  | 0.098 |
| <18 years | 54 | 9.6 | 14 | 5.2 |  |
| 18-35 years | 455 | 81.0 | 228 | 85.4 |  |
| >35 years | 53 | 9.4 | 25 | 9.4 |  |

CS Caesarean section

Missing data:

Included in the study; Weight n=6, Prematurity n=2, Place/mode delivery n=9, Mother’s age n=7 and Excluded from the study; Weight n=3, Prematurity n=3, Place/mode delivery n=5, Mother’s age n=1

| **Table S2 Summaries on Antibiotics given for the 510 neonates recruited into the study** | | | | |
| --- | --- | --- | --- | --- |
|  |  |  | **n** | **%** |
| Antibiotics given |  |  | 475 | 93.1 |
| Given ampicillin and gentamicin | |  | 455 | 89.2 |
| Treated with a third generation cephalosporin | | | 116 | 22.8 |
